# *In vitro* and *in vivo* antibacterial activities of cranberry press cake extracts alone or in combination with β-lactams against *Staphylococcus aureus*

**DOI:** 10.1186/1472-6882-13-90

**Published:** 2013-04-27

**Authors:** Moussa S Diarra, Glenn Block, Heidi Rempel, B Dave Oomah, Judy Harrison, Jason McCallum, Simon Boulanger, Éric Brouillette, Mariza Gattuso, François Malouin

**Affiliations:** 1 Pacific Agri-Food Research Center, Agriculture and Agri-Food Canada, Agassiz, BC V0M 1A0, Canada; 2 Pacific Agri-Food Research Centre, Agriculture and Agri-Food Canada, Summerland, BC V0H 1Z0, Canada; 3 Crops and Livestock Research, Agriculture and Agri-Food Canada, Charlottetown, PE C1A 4N6, Canada; 4 Département de Biologie, Centre d’Étude et de Valorisation de la Diversité Microbienne (CEVDM), Faculté des Sciences, Université de Sherbrooke, Sherbrooke, QC J1K 2R1, Canada

**Keywords:** Cranberry (*Vaccinium macrocarpon Ait*), *Staphylococcus aureus*, MRSA, Cell wall peptidoglycan, β-lactam, Synergy, Bovine mastitis

## Abstract

**Background:**

Cranberry fruits possess many biological activities partly due to their various phenolic compounds; however the underlying modes of action are poorly understood. We studied the effect of cranberry fruit extracts on the gene expression of *Staphylococcus aureus* to identify specific cellular processes involved in the antibacterial action.

**Methods:**

Transcriptional profiles of four *S. aureus* strains grown in broth supplemented or not with 2 mg/ml of a commercial cranberry preparation (Nutricran®90) were compared using DNA arrays to reveal gene modulations serving as markers for biological activity. Ethanol extracted pressed cakes from fresh fruits also produced various fractions and their effects on marker genes were demonstrated by qPCR. Minimal inhibitory concentrations (MICs) of the most effective cranberry fraction (FC111) were determined against multiple *S. aureus* strains and drug interactions with β-lactam antibiotics were also evaluated. Incorporation assays with [^3^H]-radiolabeled precursors were performed to evaluate the effect of FC111 on DNA, RNA, peptidoglycan (PG) and protein biosynthesis.

**Results:**

Treatment of *S. aureus* with Nutricran®90 or FC111 revealed a transcriptional signature typical of PG-acting antibiotics (up-regulation of genes *vraR/S*, *murZ*, *lytM*, *pbp2*, *sgtB*, *fmt*). The effect of FC111 on PG was confirmed by the marked inhibition of incorporation of D-[^3^H]alanine. The combination of β-lactams and FC111 in checkerboard assays revealed a synergistic activity against *S. aureus* including strain MRSA COL, which showed a 512-fold drop of amoxicillin MIC in the presence of FC111 at MIC/8. Finally, a therapeutic proof of concept was established in a mouse mastitis model of infection. *S. aureus*-infected mammary glands were treated with amoxicillin, FC111 or a combination of both; only the combination significantly reduced bacterial counts from infected glands (*P*<0.05) compared to the untreated mice.

**Conclusions:**

The cranberry fraction FC111 affects PG synthesis of *S. aureus* and acts in synergy with β-lactam antibiotics. Such a fraction easily obtained from poorly exploited press-cake residues, may find interesting applications in the agri-food sector and help reduce antibiotic usage in animal food production.

## Background

*Staphylococcus aureus* is an opportunistic pathogen responsible for many infectious diseases in humans and animals [1]. This bacterium is among the most prevalent causes of food poisoning gastroenteritis worldwide [2], as well as a leading cause of intramammary infections (gland inflammation, mastitis) in cows, milk quality and yield reduction, and major economic losses for the dairy industry [3]. In poultry, *S. aureus* induces infections having a major impact on the fertility and productivity of a breeder flock. Infections by *S. aureus* can occur in the joints or as a generalized infection (septicemia). Manifestations with more important economic consequences include synovitis with arthritis, osteomyelitis, and dermatitis [4]. Early efficient treatment of infection is paramount to avoid development of a chronic infection, acute infection resulting in irreversible tissue necrosis, or exacerbation leading to organ failure [5]. However, treating *S. aureus* infections becomes increasingly challenging due to its capacity to develop enhanced antibiotic resistance. Such resistance has developed due to the protective effects provided by biofilm production [6], chromosomal mutations, the acquisition or expression of various antibiotic resistance genes including those encoding low-affinity penicillin-binding proteins (PBPs) involved in cell wall peptidoglycan biosynthesis, and efflux pumps [7,8].

The first report of a penicillin-resistant *S. aureus* occurred in the 1940s, followed by the emergence of methicillin-resistant *S. aureus* (MRSA) strains, which possess the low-affinity PBP2A encoded by the *mecA* gene. These now multi-resistant strains can induce various nosocomial and community-associated infections [9]. The emergence and widespread occurrence of hospital and community acquired MRSA as well as livestock-associated MRSA resulted from antibiotic selection pressure and horizontal and vertical gene transfers [10]. Concerning livestock, a high prevalence of MRSA was reported in pigs and pig farmers [11], and in Europe, MRSA strains found primarily in pigs are now a leading cause of community-associated MRSA infections in humans [12]. Similarly, the recent emergence of bovine MRSA is also a concern since these strains are transmittable to humans [13,14]. The occurrence of MRSA among companion and food-producing animals and possible impact on human health has been reviewed by Petinaki and Spiliopoulou [15]. New research approaches, novel sources of antibiotic compounds and discovery of original microbial cell targets need to be developed in order to overcome multi-resistant bacteria such as MRSA.

The urge to bring forth novel antibiotic molecules hastened the investigation of the perspectives offered by plants. As such, many studies on berry fruits have shown the presence of large scale bioactive compounds such as cancer cell growth inhibitors, antimicrobial molecules, and antioxidants [16-18]. Cranberry (*Vaccinium macrocarpon Ait*) is one of the berries having the most potent antimicrobial effects against several human pathogens. We showed a lower mortality rate in chickens fed a commercial cranberry fruit extract (Nutricran®90) in feed compared to a non-supplemented control diet and that feeding birds diets containing cranberry fruit extract was effective in influencing the populations of bacteria such as *Enterococcus*[19]. The mechanism underlining this observation is unknown, however our study suggests that cranberry extract causes a shift in the bacterial population in the intestinal tract. Water-soluble phenolic compounds of cranberry juice, such as flavonoids (ex: anthocyanins and flavonols), have higher antimicrobial activities against foodborne pathogens than apolar phenolic compounds like polymeric tannins [16]. The bacterial cell wall and cellular membranes have been identified as targets of cranberry extracts on Gram-negative and Gram-positive foodborne pathogens like *Escherichia coli* O157:H7 and *S. aureus*[20], however, information is limited regarding the mechanism of action of cranberry on bacteria.

In this work, we have investigated the effect of Nutricran®90, and three extracts obtained from fresh cranberry fruits, on the transcriptional profiles of *S. aureus,* to provide insights on the antibacterial modes of action of such preparations. We identified a transcriptional signature that reveals inhibition of bacterial peptidoglycan biosynthesis. We also demonstrated the bactericidal effect of a combination of a cranberry extract and β-lactams *in vitro*, and the therapeutic usefulness of such a synergistic combination of agents in a mouse mastitis model of infection.

## Methods

### Bacterial strains and growth conditions

The antibiotic susceptibility testing reference strain *Staphylococcus aureus* ATCC 29213, the sequenced MRSA strains COL and N315, as well as the bovine mastitis isolates: Newbould (ATCC 29740), SHY97-4320 and SHY97-3906, were used in this study. Strains SHY97-4320 and SHY97-3906 were isolated from clinical mastitis and were previously described [21]. Bacteria stocks were kept frozen at −80°C and grown on Mueller Hinton agar or Cation adjusted Muller Hinton broth (CAMHB, Becton Dickinson, Mississauga, ON, Canada). Viable bacterial counts (CFU) were determined on Tryptic Soy agar (TSA, Becton Dickinson).

### Cranberry products, antibiotics and chemical reagents

A commercial cranberry product, Nutricran®90 (NC90), was obtained from Decas Botanical Synergies (Wareham, MA, USA). The NC90 contained at least 88% carbohydrates, 30% organic acids, 2 to 3.8% total phenolic compounds, 0.3 to 1% anthocyanins, 0.8 to 1.5% proanthocyanidins, 300 to 435 μg/g of quercetin, and was 100% soluble in water as described by the manufacturer. Laboratory and pilot-scale preparations of extracts were performed with fresh Ben Lear cranberries obtained from a local farm (Agassiz, British Columbia, Canada) or from Ocean Spray (Langley, BC, Canada) as described previously [17]. Chloramphenicol, oxacillin, amoxicillin, norfloxacin, rifampicin and vancomycin were purchased from Sigma Aldrich (Oakville, Ontario, Canada).

### Preparation of cranberry press cake extracts

Laboratory-scale fraction were obtained from 2 Kg of fresh fruits which were hammer milled through 1 cm holes then pressed in a miniature rack and cloth press at 3000 psi for 10 min to obtain juice and press cake (PC). Triplicate allotments of PC (90 ± 5 g) were placed into screw cap polyethylene centrifuge containers and covered with 95% ethanol and kept at room temperature for 48 h. Triplicate extracts were pooled, dried by rotary evaporation to remove the ethanol (Büchi rotavapor R, Brinkmann Instruments Canada Corp.; 30°C, speed 5–7, full vacuum (−25kPa; Büchi Vac V-500) with condenser temperature of 0°C), and freeze dried (Virtis 50-SRC, Gardiner, NY, USA; condenser at −56°C; vacuum at −100 milli-Torr; shelf at 25°C) to obtain fraction FC111. Replicate PC mashes were used for one and two other extractions with 95% ethanol, and the soluble fractions were freeze dried (fractions FC121 and FC131, respectively). Pilot-scale extraction procedures were previously described [17]. In brief, the fresh cranberries were pressed in a hydraulic rack and cloth press and the juice was separated from the PC. Following this step, the PC was covered with 95% ethanol for 24 h. Then, the crushed cranberries were decanted and the soluble fraction was refrigerated at 1°C (fraction FC111).

Phenolic acids, anthocyanins, flavonols and tartaric esters were determined by the method of Oomah et al. [22]. Antioxidant activity was measured through oxygen radical absorbance capacity (ORAC), as described previously [23]. The polyphenolics and antioxidant activities of the fresh cranberry fruit fractions obtained by pilot-scale ethanol extraction of the PC was previously reported [17]. As described, PC extracts including FC111 showed three to four times the phenolic acid, tartaric ester contents and antioxidant activities, as well as five to 10 times the flavonols and anthocyanins of their respective juice powders [17]. There was good similarity between the amounts of measured chemical constituents of laboratory- and pilot-scale preparations, as well as their antimicrobial activity (see result section).

### Antibiotic susceptibility testing and synergy studies

Minimal inhibitory concentrations (MICs) of antibiotics and cranberry extracts were determined by a broth microdilution technique, following the recommendations of the Clinical and Laboratory Standards Institute [24]. A checkerboard protocol [25] was conducted by a broth microdilution method similar to that used for standard MIC determinations to evaluate the effect of varying concentrations of cranberry extracts on the activity of known antibiotics against *S. aureus* strains. The fractional inhibitory concentration (FIC) indices were calculated as follow: FIC index = FIC_A_ + FIC_B_ = A/MIC_A_+ B/MIC_B_, where A and B are MICs of compounds A and B in combination, MIC_A_ and MIC_B_ are the MICs of compound A and compound B alone and FIC_A_ and FIC_B_ are the FICs of compound A and of compound B. Indifference for drug interactions or an additive effect is demonstrated if the FIC index is >0.5-4, a synergy if the FIC index is ≤ 0.5, whereas an antagonistic effect is represented by a FIC index of >4 [26].

### Time-kill experiments

Time-kill experiments were performed to observe the bactericidal effect (*i.e*., a 3 log10 reduction in CFU/mL) of cranberry extracts alone or in combination with sub-inhibitory concentrations (sub-MICs) of traditional antibiotics. The CAMHB medium was inoculated with *S. aureus* at ~10^5^-10^6^ CFU/mL and grown at 35°C, with shaking (225 rpm) in the absence or presence of tested agents at the specified concentrations (see the figure legends). At several time points, bacteria were sampled, serially diluted and plated on TSA for CFU determinations.

### Transcriptional profiles

The modulation of transcriptional profiles of *S. aureus* strains ATCC 29213, MRSA N315, SHY97-4320 and SHY97-3406 by the cranberry product NC90 was first evaluated by exploratory transcriptional profiling experiments using a sub-genomic array as described previously (in detail) [27,28]. Bacteria (~10^7^-10^8^ CFU/mL) were exposed to NC90 at the MIC (2 mg/mL) for 30 min in CAMHB. Culture samples (~10^8^ cells) were treated with RNAprotect (Qiagen, Mississauga, ON, Canada) for at least 10 min. Bacterial pellets were then suspended in 100 μL of TE buffer containing 200 μg/mL lysostaphin and incubated at room temperature for 1 h. RNA was extracted using the RNeasy Mini Kit and treated with the RNAse-free DNAse set (Qiagen). Total RNA (2.5 μg) and 1 μL of the appropriate RNA spike from Lucidea Universal Scorecard (GE Healthcare Life Sciences, Baie D’Urfé, QC, Canada) was reverse transcribed using 5 μg of random hexamers, a dNTP mix, 5-[3-aminoallyl]-2-dUTP and the Superscript II RT (Invitrogen, Life Technologies Inc., Burlington, ON, Canada). The amino-modified cDNA was purified and labeled with NHS-Cy3 (untreated control) and -Cy5 (bioactive agent test) prior to hybridization on GAPS II slides (Corning, NY, USA). The probes (200 pmol, ≥8% incorporation) were suspended in 18.5 μL of hybridization buffer (5X SSC, 0.1% SDS, 25% formamide, 100 μg/ml mouse COT1 DNA (Invitrogen)). The prehybridization, hybridization and washing steps were done as prescribed for Corning Gaps II Slides. Hybridization signals for each spot were quantified with the ScanArrayExpress Microarray Scanner and the ScanArrayExpress software V 2.2.0.0022 (Perkin Elmer, Wellesley, MA, USA). Intensity of each dye was adjusted using the signal of the control spots from Lucidea Universal Scorecard and data were submitted to the Lowess normalization. Only signals showing intensity three times above the background were analyzed as described previously [18,27]. Expression ratios (Cy5 [treated]/Cy3 [untreated control]) were represented in log2. Only log2 ratios with a minimum mean of ≥2.0 (or ≤ −2.0) obtained for at least two of the four *S. aureus* strains tested were considered. Data represent the average of triplicate spots from duplicate independent experiments for each of the four tested *S. aureus* strains. The modulation of expression of marker genes providing transcriptional signatures was thereafter confirmed by qPCR analyses.

### Quantitative PCR (qPCR)

Transcriptional modulations induced by cranberry fractions and observed by using exploratory microarrays were confirmed by qPCR for a selection of marker genes and strains. Additional RNAs, from three independent bacterial cultures and prepared as described above, were obtained for qPCR analyses. The qPCR assays using SYBR green (Invitrogen) and the JumpStart Taq DNA Polymerase (Sigma) were performed as described previously [27] for the marker *vraS*, *sgtB*, *murZ*, *mrsA*, *lytM* and *mntB* genes indicative of a cell wall stress [29,30]. The relative expression ratios were calculated using the cycle threshold (*C*_t_) of the untreated culture (no-bioactive agent control) and that of the housekeeping *gyrA* gene [n-fold expression = 2^-ΔΔ*C*t^, where ΔΔ*C*_t_ = Δ*C*_t_ (treated culture)/Δ*C*_t_ (untreated culture) and Δ*C*_t_ represents the difference between the *C*_t_ of the gene studied and the *C*_t_ of *gyrA*]. The sequences of primers for qPCR were: *gyrA*-forward 5^′^-CATTGCCAGATGTTCGTGAC -3^′^ and *gyrA*-reverse 5^′^-CACCAACGATACGTGCTGAT -3^′^; *vraR-*forward AAAAGATATCGCCGATGCAG and *vraR-*reverse ATAACTCTGCCGCGCTTTTTC; *sgtB-*forward AAACCGCCGAAAAAGAAAAA and *sgtB*-reverse TCATCCACATTATCGCGTGT; *msrA-*forward GCAAATGGTGTAGGTAAGACAACT and *msrA*-reverse ATCATGTGATGTAAACAAAAT; *murZ-*forward ACGCACACTAAATGGGGAAG and *murZ*-reverse CCTCAGCAATTAAACCAGCA; *lytM-*forward ATTAACAGCAGCAGCGATTG and *lytM-*reverse TGTGCTTGTTGGGTGTTTGT; *mntB-*forward CCTGGTGTTGCCCTATCATT and *mntB*-reverse GGCGTCAGGTTTCGTTTTAC.

### Inhibition of macromolecular biosynthesis

Macromolecular biosynthesis assays were performed exactly as described previously [31]. Briefly, *S. aureus* ATCC 292123 was grown in a complete defined medium, and transferred to a round-bottom microwell plate containing radiolabeled precursors [^3^H]-Leucine; [^3^H]-Thymine; [^3^H]-Uridine (Perkin Elmer, Montreal, QC, Canada) or D-[^3^H]-Alanine (American radiolabeled chemicals, St-Louis, MO, USA), as well as various dilutions of antibiotic agents. Incorporation was allowed to proceed for 45 min (for Leu and D-Ala), or 35 min (for Thy and Uri) at 35°C. The reaction was terminated by transferring the mixtures into pre-chilled trichloroacetic acid (10% final), followed by collection of the precipitated macromolecules on GF/C filter using a dot-blot filtration system. The dried filter was cut and the level of radiolabeled precursor incorporation was determined by liquid scintillation counting. Data represent the average of three independent experiments.

### Mouse mastitis model of infection

The mastitis model was previously optimized for experimental therapy [32]. CD-1 lactating mice (Charles River, St-Constant, Canada) were used 12 to 14 days after offspring birth. Pups were removed 1 h before intramammary inoculation, under anesthesia, of both L4 (on the left) and R4 (on the right) abdominal mammary glands with 100 μL of *S. aureus* Newbould (1 CFU/μL). Glands were treated (intramammary) 4 h after infections with amoxicillin, a cranberry extract or a combination of both. Mice were then sacrificed 14 h post-treatment for mammary gland sampling, homogenization, plating, and bacterial counts. Data represent two independent experiments using several mice and glands in each.

The animal experiments were conducted following the guidelines of the Canadian Council on Animal Care. Protocol 08-FM1 was specifically approved by the institutional ethics committee on animal experimentation of the *Faculté des Sciences of Université de Sherbrooke* to perform the animal studies included in the present article.

### HPLC-MS analysis of cranberry fraction FC111

One milligram aliquots of each dry powdered fraction were suspended in 20% aqueous methanol, 0.1% TFA, at 1 mg/mL, prior to HPLC-MS analysis. This analysis was performed on an Agilent 1100 series LC/MSD SL system (Agilent Technologies, Mississauga, ON), equipped with a quaternary pump, in-line degasser, robotic auto sampler and diode array (DAD) and single quadropole mass spectrometry (SQD) detectors. The separation and characterizations were achieved using a Zorbax Eclipse XDB-C18 (Agilent Technologies) analytical column (4.6 × 150 mm, 5 μm particle size) fitted with a Zorbax Eclipse XDB-C18 analytical guard column (4.6 × 12.5 mm, 5 μm particle size). A tertiary solvent system was used, (A = 0.1% aqueous trifluoroacetic acid; B = water:methanol:trifluoroacetic acid 1:1:0.1; C = acetonitrile), at a flow rate of 0.5 mL/min, with a column temperature of 45°C, and 15 μL sample injection volume , according to the following gradient: Time = 0 min. A:B = 100:0; Time = 2 min. A:B = 85:15; Time = 20 min. A:B = 0:100; Time = 26 min. B = 100% isocratic; Time = 30 min. B:C = 0:100; Time = 33 min. C = 100% isocratic; post time equilibration (3 min.) A = 100%. The entire UV–vis absorption spectrum (190–900 nm) was recorded, while general phenolics (280 nm), anthocyanins (520 nm), and flavonols (360 nm) channels were monitored. Atmospheric pressure electrospray ionization mass spectra in both positive (PIM) and negative (NIM) ion modes (PIM = 150–1400 amu; NIM = 150–2000 amu) were recorded on duplicate injections of the same sample (drying gas flow 10 L/min; nebulizer pressure 30 psig; dry gas temperature 350°C; PIM capillary voltage 3000; NIM capillary voltage 3500). Peak identities were assigned using a combination of UV–vis absorption spectra, MS fragmentation patterns, congruent retention times with commercially available standards, and comparison to relevant cranberry literature [33-35].

### Statistical analysis

Statistical analyses were carried out with the GraphPad Prism Software (v.5.00). Tests used for the analysis of each experiment are specified in the figure legends.

## Results

### The NC90 and the PC fraction FC111 are bactericidal and affect the transcription of genes associated with a cell wall stress

The MIC of the cranberry product NC90 against *S. aureus* ATCC 29213, Newbould and MRSA COL was 2 mg/mL. In an attempt to understand the basis of the growth inhibitory effect of NC90 observed on *S. aureus*, we studied the modulation of bacterial gene expression for a variety of strains by microarray analyses (Table 1). Exposure of any of the four tested *S. aureus* strains (ATCC 29213, MRSA N315, SHY97-4320, SHY97-3906) to NC90 up-regulated strongly several genes involved in cell wall biosynthesis, similar to the up-regulation of genes caused by antibiotics known to disrupt peptidoglycan biosynthesis. Indeed, the strong expression of *fmt*, *pbp2*, *murZ*, *sgtB*, *tcaA* and *vraS/R* genes is evidence of a cell wall stress induced by the tested cranberry product NC90 (Table 1). Our data are consistent with the results from other laboratories on the transcriptional profiles obtained by exposure of *S. aureus* to oxacillin, bacitracin and D-cycloserine [30] or to daptomycin and vancomycin [29] and these similarities are also reported in Table 1. NC90 also up-regulated *lytM*, encoding a peptidoglycan hydrolase distinctively like daptomycin (Table 1), and down-regulated genes such as *spa*, *mntABC*, *isdA*, *hla*, and *femC*.

**Table 1:** **Genes up- and down-regulated for at least two of four *S. aureus *strains following a 30-min exposure to the cranberry product NC90 (2 mg/mL)**

**ORF SACOL**	**Gene name**	**Gene annotation**	**Fold expression**^**a **^**for each strain**								
			**ATCC 29213**	**MRSA N315**	**SHY97 4023**	**SHY97 3906**	**DAP**^**b**^	**VAN**^**b**^	**OXA**^**b**^	**OBC**^**c**^	**Vra**^**d**^
**Up-regulated genes**											
0263		Peptidoglycan hydrolase	15.0	24.5	3.6	9.8	+	=	=	=	
1066	*fmt*	Fmt protein	6.8	3.8	2.9	2.0	+	+	+	+	+
1459		Methionine sulfoxide reductase	2.3	6.0	−1.5	1.2	=	+	+	+	
1490	*pbp2*	Penicillin binding protein 2	2.5	2.8	1.0	1.9	+	+	+	+	+
1777	*htrA*	Putative serine protease Htra	4.6	8.9	1.0	2.1	+	+	+	=	
1932		Transglycosylase domain protein	8.5	6.8	2.2	2.5	+	=	=	+	+
1942	*vraR*	DNA-binding response regulator	8.8	10.9	nd	2.7	+	+	+	=	+
1943	*vraS*	Sensor histidine kinase VraS	14.2	10.5	7.6	4.0	+	+	+	+	+
1944		Hypothetical protein	5.1	7.5	1.3	1.4	+	+	+	+	+
2017	*groES*	Chaperonin	nd	2.2	2.3	2.2	+	+	+	=	
2116	*mur*AB	UDP-N-AcGlc-1-carbotransferase	4.0	7.6	2.5	2.6	+	+	+	+	+
2291		Staphyloxanthin biosynthesis	6.0	2.2	4.5	3.9	–	–	–	=	
2352	*tcaA*	TcaA protein	5.1	6.3	1.1	1.4	+	+	+	+	+
2584	*isaA*	Immunodominant antigen A	2.1	1.8	2.3	2.1	=	=	=	=	
**Down-regulated genes**											
0095	*spa*	IgG binding protein	−33.3	−2.0	−37.5	nd	–	=	=		
0096	*sarS*	Accessory regulator	−2.0	−2.9	−2.7	−2.2	–	–	–		
0136-0150^e^	*capA-O*	Capsular polysaccharide	−4.9	nd	−3.1	−1.9	–	=	–		
0317		Lipase precursor	−8.6	−6.8	−4.1	−1.7	–	–	=		
451-452^e^	*ahpFC*	Alkyl hydroperoxide reductase	−3.4	−3.6	−8.2	−1.2	=	=	–		
0679		Na+/H+ antiporter, MnhA	−4.5	−10.0	−13.0	−25.0	=	=	=		
0688-0690^e^	*mntABC*	ABC transporter	−43.5	−66.7	−86.7	−91.7	–	=	=		
0765-0766^e^	*saeSR*	Sensor/Histidine kinase	−1.5	−3.7	−1.5	−3.6	=	=	=		
1072	*folD*	THF dehydrogenase	−2.1	−2.6	−2.7	nd	=	=	=		
1079	*purF*	amidophosphoribosyltransferase	nd	−10.0	−0.8	−2.6	=	–	–		
1140	*isdA*	Heme-iron transport	−5.3	−2.1	−2.9	nd	–	=	=		
1158-1160^e^	*sdhABC*	Succinate dehydrogenase	−5.1	−7.9	−4.5	−3.8	–	–	=		
1173	*hla*	Alpha-hemolysin	−3.6	−4.3	nd	−2.6	–	=	=		
1262-1263^e^	*sucCD*	Succinyl-CoA synthetase	−5.6	−3.1	−6.4	−2.3	=	=	=		
1328	*glnR*	Glutamine synthetase repressor	−3.7	−6.3	−3.4	−5.3	–	–	=		
1329	*femC*	Glutamine synthetase FemC	−2.7	−4.2	−3.2	−2.9	–	=	=		
1368	*katA*	Catalase	−3.2	−2.4	−5.2	−2.1	=	+	=		
1385	*acnA*	Aconitate hydratase	−3.4	−3.0	−6.3	nd	=	=	=		
1396	*fmtC*	FmtC protein	−0.9	−2.4	−2.0	nd	–	–	–		
1448-1449^e^	*sucAB*	2-oxoglutarate dehydrogenase	−8.1	−5.4	−4.8	−4.9	=	=	=		
1741	*icd*	Isocitrate dehydrogenase, NADP	−5.6	−2.2	−11.5	−2.5	=	=	+		
1742	*gtlA*	Citrate synthase	−6.7	−2.3	−14.3	−2.2	=	=	+		
1743		Amino acid permease	−3.1	−1.2	−4.8	−1.5	=	=	=		
2055	*rsbW*	Anti-sigma B factor	−2.6	nd	−1.6	−2.1	–	=	–		
2057	*rsbU*	Sigma factor B regulator protein	−1.3	−3.1	−0.9	−2.1	=	=	–		
2066-2068^e^	*kdpABC*	Potassium-transporting ATPase	−3.5	nd	−2.3	−1.7	=	=	=		
2293		NAD/NADP dehydrogenase	−2.0	−2.4	−1.3	nd	–	=	–		
2694	*geh*	lipase	−27.3	−4.3	−20.0	nd	=	=	=		

^a^ Data are expressed in log2 ratios of Cy5(cranberry-treated)/Cy3(untreated control); see material and methods.

^b^ Up (+) or down (−) expression of the indicated gene by exposure to daptomycin (DAP), vancomycin (VAN) or oxacillin (OXA), as reported by Muthaiyan et al. [29]; the equal sign (=) indicates genes that were not affected.

^c^ Up (+) or down (−) expression of the indicated gene by exposure to oxacillin (O), bacitracin (B) and D-cycloserine (C) as reported by Utaida et al. [30]; the equal sign (=) indicates genes that were not affected.

^d^ The plus sign (+) indicates genes known to be regulated by the two-component response regulator VraSR following a cell wall stress [42].

^e^ To avoid repetition, the fold modulation of genes arranged in operons is represented as an average for the indicated gene set.

To follow up, extracts from press cakes (PC) were investigated. The MIC of FC111 (lab- and pilot scale extracts) was lower than that of NC90 and represented 1 mg/mL against *S. aureus* ATCC 29213, Newbould and MRSA COL. Besides, the MIC value for both FC121 and FC131 was higher (3 mg/mL). Since FC111 showed the lowest MIC (best efficiency), its bactericidal effect against *S. aureus* ATCC 29213 and MRSA COL was evaluated in time-kill experiments. FC111 at 1 mg/mL (MIC) induced more than a 3-log10 reduction of CFU/mL against both strains compared to their respective untreated control cultures after 8 h of incubation and a concentration of 2 X MIC (2 mg/mL) yielded more than a 6-log10 reduction of CFU/mL at 8 h and no detectable CFU after 24 h (data not shown).

**Figure 1:**
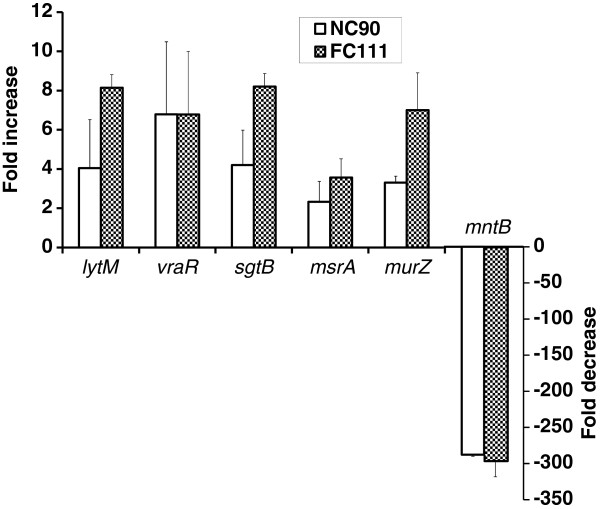
**qPCR analysis of transcriptional modulations induced in *S. aureus *strain ATCC 29213 by the cranberry product NC90 or fraction FC111.**

Subsequently, we used qPCR to first confirm the transcriptional effects induced in *S. aureus* ATCC 29213 following exposure to NC90 in DNA array experiments (above) and secondly, to detect similar transcriptional modulations induced by the most active ethanol-extracted PC fraction FC111. Modulation of the same cell wall stress markers (*lytM*, *vraR*, *sgtB*, *msrA*, *murZ* and *mntB*) was indeed detected for both FC111 and NC90, indicating a similar mode of action against *S. aureus* (Figure 1).

### FC111 inhibits cell wall biosynthesis

**Figure 2:**
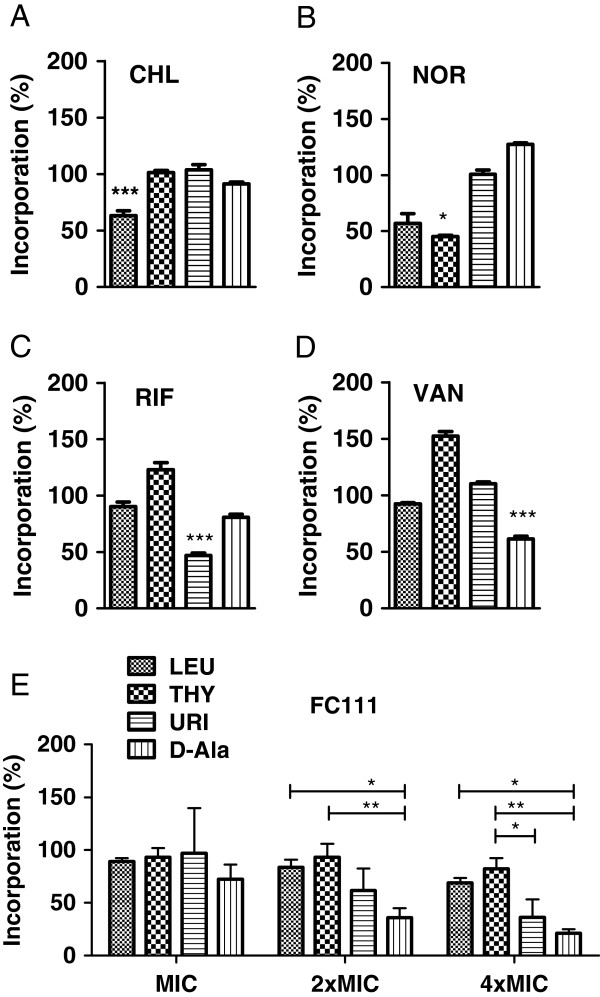
**Comparison of the inhibitory effect of a variety of molecules on macromolecular biosyntheses in *S. aureus *ATCC 29213.** Chloramphenicol (Panel **A**, CHL), norfloxacin (Panel **B**, NOR), rifampicin (Panel **C**, RIF), vancomycin (Panel **D**, VAN), and the cranberry fraction FC111 (Panel **E**). For **A**-**D**, a one-way analysis of variance (ANOVA) with Dunnett’s post test was performed where the macromolecule’s biosynthesis associated to the test antibiotic served as comparator for the three others (*, *P* <0.05; ***, *P*<0.001). In **E**, a two-way ANOVA with Bonferroni’s post test was performed (*, *P*<0.05; **, *P*<0.01).

Whole-cell macromolecular biosynthesis assays were performed to elucidate the mechanism of action of FC111 and to confirm its inhibitory effect on cell-wall biosynthesis. *S. aureus* ATCC 29213 was treated with several concentrations of the cranberry fraction FC111 or known antibiotics and the percentage of incorporation of radiolabeled precursors into macromolecules was evaluated (Figure 2). All known drugs targeted the expected macromolecular biosynthesis; chloramphenicol, norfloxacin, rifampicin and vancomycin inhibited protein, DNA, RNA and cell wall biosynthesis, respectively. In the presence of 2 (2 mg/ml) and 4 times (4 mg/ml) the MIC of the cranberry fraction FC111, the incorporation of D-[^3^H]Ala, a precursor of cell wall peptidoglycan, was significantly reduced compared to that observed for precursors of DNA, RNA and proteins (Figure 2E).

### *In Vitro* bactericidal effect of FC111 and β-lactam antibiotic combinations

Since exposure of *S. aureus* to FC111 induced cell wall stress, we investigated the potential synergy between this cranberry PC fraction with amoxicillin (used for treatment of *S. aureus*-induced bovine mastitis in veterinary medicine) and oxacillin (used for many clinical indications in humans). Checkerboard assays were performed and a FIC index was determined for each tested combination against *S. aureus* ATCC 29213 (β-lactam susceptible strain) and MRSA COL (with the *mecA*/PBP2A-mediated β-lactam resistance). The cranberry fraction FC111 increased the inhibitory activity of amoxicillin and oxacillin against both tested *S. aureus* strains by triggering up to 512-fold reductions in β-lactam MIC values (Table 2). Combination of FC111, 128 or 256 μg/mL (*i.e*., 1/8 or 1/4 FC111 MIC, respectively), with either of the β-lactams yielded FIC indices of ≤0.25, for each of the strains tested. These FIC indices clearly showed synergy between FC111 and β-lactams against *S. aureus*. Data also demonstrated that this synergy can efficiently bypass the *mecA*-mediated mechanism of β-lactam resistance found in MRSA strain COL.

**Table 2:** **Minimal inhibitory concentrations (MIC) of amoxicillin and oxacillin alone or in combination with increasing concentrations (0 to 256 μg/ml) of the cranberry PC fraction FC111 against *S. aureus *ATCC 29213 and MRSA COL**

***S. aureus *strains**	**Antibiotic**	**MIC of antibiotic in μg/mL in the presence of an increasing concentration of the cranberry fraction FC111, also in μg/mL (fold decrease in antibiotic MIC)**^**a**^				
		**0**	**32**	**64**	**128**	**256**
ATCC 29213	amoxicillin	0.06	0.06 (1×)	0.06 (1×)	0.015 (4×)	0.015 (4×)
MRSA COL		512	4 (128×)	4 (128×)	1 (512×)	1 (512×)
ATCC 29213	oxacillin	0.5	0.06 (8×)	0.03 (16×)	0.03 (16×)	0.03 (16×)
MRSA COL		512	256 (2×)	128 (4×)	32 (16×)	16 (32×)

^a^ Combination of 128 or 256 μg/mL of FC111 with either antibiotic yielded FIC indices of ≤0.25 against all tested strains.

**Figure 3:**
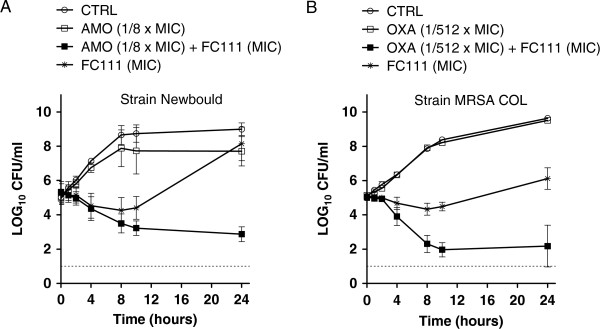
***S. aureus *kill-curve kinetics evaluated in cation-adjusted Mueller Hinton broth containing FC111 alone or in combination with the specified antibiotic.** Panel **A**, FC111 alone (at its MIC, 1 mg/mL) or in combination with a sub-MIC (1/8 x MIC) of amoxicillin against *S. aureus* strain Newbould, and Panel **B**, with a sub-MIC (1/512 x MIC) of oxacillin against MRSA COL. Values are means of duplicates of two separate experiments. Bars represent standard deviations (SD).

The synergistic effect arising from the combination of β-lactams and FC111 in checkerboard assays was explored in greater details by using kill kinetic studies. Combination of the cranberry fraction FC111 at its MIC (1 mg/mL) and 1/8 × MIC of amoxicillin induced a strong bactericidal effect (>3 Log10 CFU/mL) against a bovine mastitis strain (*S. aureus* Newbould, also used in our mastitis model of infection in the mouse [see below]) compared to the bactericidal effect observed with FC111 or amoxicillin alone (Figure 3A). Moreover, the combination of FC111 at its MIC and oxacillin (1/512 × MIC) also showed strong bactericidal effect against *S. aureus* MRSA COL (Figure 3B).

### The FC111-amoxicillin combination improves bacterial clearance in mammary glands

**Figure 4:**
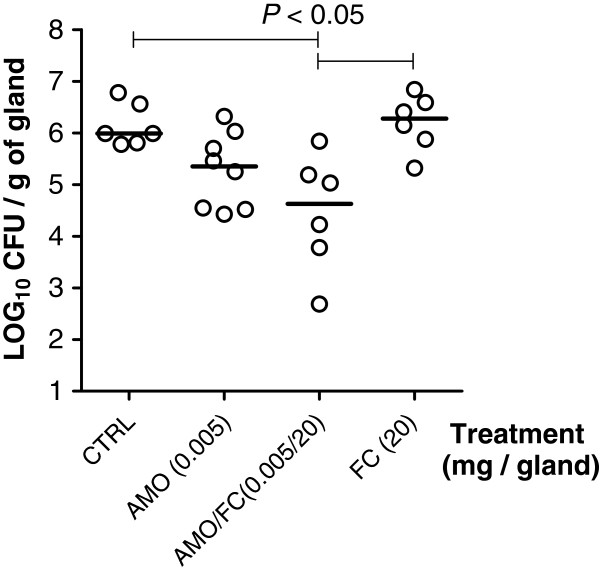
**Bacterial counts (CFU) obtained from mice mammary glands 10h post-infection with *S. aureus.*** Mice mammary glands were treated (intra-mammary administration) 4h after infection with PBS with or without amoxicillin (AMO), the cranberry fraction FC111 (FC) or a combination of both at the indicated concentration (mg/mL). Each dot represents the CFU of each individual gland (n= 6–8) and the median value for each group is indicated by the bar. Statistical differences (*P*<0.05) between CFU recovered from treated and untreated animals are shown (non-parametric Kruskal-Wallis ANOVA with Dunn's post test).

The *in vivo* relevance of the synergy between the cranberry extract FC111 with β-lactams was determined using a mouse mastitis model. The mice were infected with the bovine strain *S. aureus* Newbould and treated with FC111, amoxicillin or a combination of both (Figure 4). Compared to the untreated mice, there was no significant change in bacterial loads (CFU/g of gland) when mice were treated with either one of the compounds. However, a significant reduction in bacterial counts (1.8 Log10 CFU/g of gland) was observed using the FC111-amoxicillin combination.

### HPLC-MS analysis of cranberry fraction FC111

**Figure 5:**
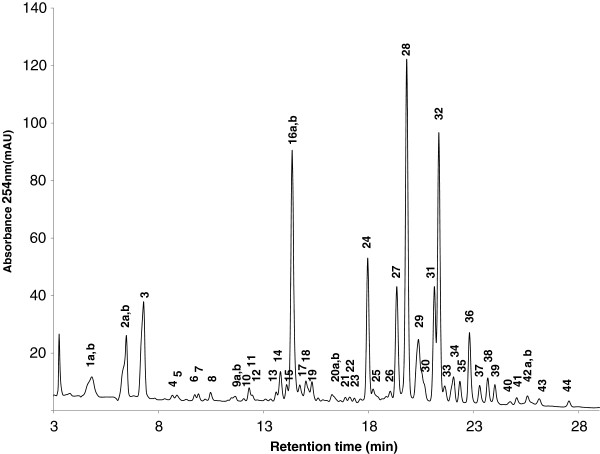
**C18 reversed-phase HPLC chromatogram of laboratory scale fraction FC111, displaying separation of 44 constituents.** Retention time is in minutes, and absorbance measured at 254 nm (mAU). Alpha numeric labels refer to peaks discussed in the main text, and summarized in Table 3.

HPLC-MS was performed on laboratory-scale cranberry fraction FC111, which had the following spectrophotometrically measured [22] classes of chemical compounds per gram of freeze-dried powder: 132.4 (total phenolics), 8.3 (tartaric esters), 9.6 (flavonols) and 23.8 (anthocyanins) mg equivalents of catechin, caffeic acid, quercetin and cyanidin-3-glucoside, respectively. The total phenolics, tartaric esters and flavonols compared well with the composition of the pilot scale sample FC111 [17]. Laboratory- and pilot-scale prepared FC111 provided identical antibacterial activity against *S. aureus* strains as reported above. HPLC separation of cranberry fraction FC111 constituents was achieved in less than 30 min, using the program described above. A typical chromatographic trace, recorded at 254 nm from laboratory scale FC111 (Figure 5), demonstrates the separation of iridoids, hydroxybenzoic acid, hydroxycinnamic acid, anthocyanin and flavonol components (Table 3). Compounds lacking UV-absorbing chromophores (sugars, small organic acids etc.) were omitted from analysis. In fraction FC111, qualitatively representative of all PC extracts, the principal components were anthocyanins, peaks 21, 24–29, 31, 33 (40%), flavonols, peaks 30–32, 34–44 (25%), iridoids, peaks 2a,b, 3, 16b (22%), and the conjugated glycosides of simple phenolic acids, peaks 4–15, 16a, 17–19 (9%), based on integrated peak areas recorded at 254 nm measured against total absorbance. Absolute quantification of peaks (mass balance) could not be measured, due to lack of analytical standards. Relative abundances between classes of molecules were calculated however, using integral values measured at 254 nm, while specific wavelengths were used to compare individual compounds within a class; 520, 360, and 280 nm for anthocyanins, flavonols and phenolics, respectively (Table 3, peak identities). The primary anthocyanins present were peonidin-3-O-galactoside (peak 28, 43% abundance), cyanidin-3-O-galactoside (peak 24, 18% abundance) and peonidin-3-O-arabinoside (peak 31, 15% abundance) (Table 3, abundances).

**Table 3:** Laboratory scale cranberry fraction FC111 constituents separated and characterized by HPLC-DAD-MS.

**Peak**	**t**_**R **_**(min)**^**a**^	**λmax (nm)**^**b**^	**MW (Da)**^**c**^	**Putative identification**	**Relative abundance**^**d**^
1a,b	4.8		192	quinic acid	
**Iridoids**					
2a,b	6.5	235	390	monotropein	18.5 ± 0.78%
3	7.3	235	392	6,7-dihydromonotropein	26.8 ± 0.07%
16b	14.4	235	416	unknown monotropein derivative	54.8 ± 0.99%
				Total (vs. A254 nm)	22 %
**Monophenolic acids**					
4	8.7	280	220	unknown benzoic acid derivative	3.1 ± 0.51%
5	8.9	280		unknown benzoic acid derivative	0.52 ± 0.06%
6	9.7	290	162	unknown benzoic acid derivative	0.78 ± 0.10%
7	9.8	290		unknown benzoic acid derivative	1.18 ± 0.11%
8	10.4	275	316	dihydroxybenzoic acid glycoside	3.45 ± 0.29%
9a,b	11.6	285, 320	342	caffeic acid glycoside	3.06 ± 0.45%
10	12.0	275	300	hydroxybenzoic acid glycoside	0.72 ± 0.16%
11	12.3	295, 305	326	coumaric acid glycoside isomer 1	14.04 ± 0.49%
12	12.4	275	238	unknown benzoic acid derivative	1.45 ± 0.049%
13	13.6	315	326	coumaric acid glycoside isomer 2	15.1 ± 0.40%
14	13.8	285, 310	326	coumaric acid glycoside isomer 3	16.1 ± 0.56%
15	14.1	295, 325		unknown hydroxycinnamic acid	7.22 ± 0.092%
16a	14.4	280	284	benzoyl-glycoside	5.44 ± 0.89%
17	14.7	285, 330	356	ferulic acid-glycoside	5.59 ± 0.21%
18	15.0	275		unknown benzoic acid derivative	13.24 ± 0.13%
19	15.3	280, 330	386	sinapinic acid-glycoside	7.47 ± 0.28%
20a,b	16.3	280		unknown benzoic acid derivative	1.50 ± 0.69%
				Total (vs. A254 nm)	9%
**Anthocyanins**					
21	16.8	525	465	delphinidin-3-O-galactoside	0.40 ± 0.00%
24	18.0	518	449	cyanidin-3-O-galactoside	18.4 ± 0.01%
25	18.2	525	435	delphinidin-3-O-arabinoside	0.60 ± 0.03%
26	19.0	515	433	pelargonidin-3-O-galactoside	0.28 ± 0.00%
27	19.3	520	419	cyanidin-3-O-arabinoside	11.2 ± 0.00%
28	19.8	520	463	peonidin-3-O-galactoside	43.3 ± 0.03%
29	20.4	524	493	malvidin-3-O-galactoside	7.64 ± 0.00%
31	21.1	521	433	peonidin-3-O-arabinoside	15.5 ± 0.01%
33	21.6	530	463	malvidin-3-O-arabinoside	1.79 ± 0.00%
				Total (vs. A254 nm)	40%
**Flavonols**					
30	20.5	370	450	myricetin-3-O-arabinoside	4.05 ± 0.34%
32	21.3	360	464	quercetin-3-O-galactoside	50.2 ± 0.11%
34	22.0	354	434	quercetin-3-O-xyloside	7.71 ± 0.00%
35	22.3	352	434	quercetin-3-O-arabinopyranoside	3.72 ± 0.02%
36	22.8	354	434	quercetin-3-O-arabinofuranside	13.65 ± 0.12%
37	23.3	352	448	quercetin-3-O-rhamnoside	2.87 ± 0.01%
38	23.6	356	478	isorhamnetin-3-O-galactoside	5.31 ± 0.04%
39	24.0	360	508	syringetin-3-O-galactoside	4.70 ± 0.05%
40	24.7	360		unknown flavonol derivative	0.46 ± 0.02%
41	25.0	355	448	isorhamnetin-3-O-arabinoside	1.55 ± 0.01%
42a,b	25.6	355	610	quercetin-3-O-(6”coumaroyl)-galactoside	2.27 ± 0.04%
43	26.1	355	478	syringetin-3-O-arabinoside	2.04 ± 0.04%
44	27.5	356	568	quercetin-3-O-(6” benzoyl)-galactoside	1.48 ± 0.02%
				Total (vs. A254 nm)	25%

^a^ t_R_ , retention time of peak (measured in minutes).

^b^ λmax, refers to characteristic UV–VIS absorption maxima (nm) pertinent to identification.

^c^ MW, molecular weight (measured in Daltons).

^d^ Relative abundance reported as percentage with associated standard deviation (based on two separate HPLC runs); iridoids calculated vs. 254 nm; monophenolic acids calculated vs. 280 nm; anthocyanins calculated vs. 520 nm; flavonols calculated vs. 360 nm.

## Discussion

The outbreak of multidrug resistant bacteria arose from several factors, including the lack of novelty in the pipeline of drug discoveries since the 1980s, and the large and non-adequate administration of antibiotics [7,36]. To eradicate this problem, explorations of new sources of antimicrobial compounds, and/or finding ways to strengthen the action of well-known antibiotics are needed [31,37]. For many decades, cranberry juice and extracts have been studied for their antimicrobial potential against bacterial pathogens. Cranberry constituents are known to exert anti-adhesion and antimicrobial activities against several pathogenic bacteria and have been suggested to prevent urinary tract infections caused by uropathogenic P-type *Escherichia coli*[38-40]. However, the mechanisms supporting the diverse effects of cranberry on microbes are poorly understood. In this study, we produced cranberry extracts by mechanical and ethanol extraction, investigated the antibacterial modes of action of these extracts by transcriptional and macromolecular biosynthesis analyses, and assessed their *in vitro* and *in vivo* bactericidal activities.

In an attempt to investigate the mode of action of the cranberry product NC90, we initiated the study by performing an exploratory transcriptional analysis by microarray, which yielded the identification of several bacterial genes known to be up-regulated in the presence of cell-wall acting antibiotics, such as oxacillin, vancomycin and daptomycin [29,30,41]. Also, the cell wall stress stimulon we observed correlated well to the list of VraS/R-regulated genes known to be up-regulated in response to the inhibition of cell wall biosynthesis [42], as reported in Table 1. Interestingly, the cranberry PC extract FC111 showed a slightly lower MIC than the commercial preparation NC90 against *S. aureus*, indicating enrichment in some antibacterial compounds in FC111. In addition, qPCR analysis of key cell wall stress markers (*vraR/S*, *sgtB*, *msrA*, *murZ*) revealed similar gene expression modulations by both NC90 and the fraction FC111, indicating that the growth inhibitory compounds remaining in FC111 should be responsible for the cell wall stress observed following exposure of *S. aureus* to this extract.

Although oxacillin, vancomycin and daptomycin disrupt cell wall peptidoglycan synthesis, these antibiotics act in different ways [29,43]. In the present study, it was interesting to observe that NC90 and FC111 also modulated the expression of *lytM* and the *mntABC* operon reacting to membrane depolarization, as distinctively reported for daptomycin by Muthaiyan et al. [29]. This may indicate that NC90 and the cranberry extract FC111 induce *S. aureus* membrane depolarization, as that observed after exposure to daptomycin. The gene modulations induced by cranberry also support the *S. aureus* cell disruption reported by Wu et al. [20], while, scanning electron microscopy of MRSA after treatment with cranberry proanthocyanidins induced negligible effect on cell morphology [44].

The action of our cranberry extract on cell wall biosynthesis was also confirmed by macromolecular biosynthesis assays in which incorporation D-Ala into cell wall materials was most affected. Hence, it was therefore tempting to conduct synergy assays with other cell-wall active antibiotics. Because β-lactams are widely used in human and veterinary medicine and because their activity is often impaired by bacterial mechanisms of resistance such as the presence of β-lactamases or the *mecA* gene in MRSA strains, we estimated that such a class of antibiotic compounds may greatly benefit from the action of cranberry in a variety of clinical applications. Our results showed that the combination of FC111 and amoxicillin or oxacillin used at sub-MICs, is highly bactericidal against either β-lactam-susceptible or -resistant MRSA strains. Moreover, this synergy was proven effective in a murine model of *S. aureus*-induced mastitis.

Resistance is part of the microbial evolution process and the use of antibiotics in human or animals applies a selective pressure favoring resistance to emerge. We did indeed report the presence of multi-antibiotic-resistant bacteria in animal food production [45,46]. In order to reduce antibiotic use, which could help break down emergence of antibiotic resistance in conventional and organic animal production, several alternatives to antibiotics have been investigated although none of these alternatives was proven to be as efficient as antibiotics [47,48]. We previously suggested that cranberry fruit derivatives could be developed to improve health and on-farm food safety while reducing the use of antibiotics as growth promoters [19]. It was recently suggested that fruit pomace can be utilized as a good source of inexpensive antioxidants for improving human health and reducing the risks of some chronic diseases [49]. Recently, we showed that our FC111 fraction from cranberry pomace (press cake) is an excellent natural polyphenolics product with increased antioxidant and vasorelaxant benefits [17]. In the present study, we demonstrated that this FC111 fraction may be developed as a viable alternative to traditional antibiotics or at least to significantly potentiate their activities against multidrug resistant bacteria such as MRSA, and thus, to significantly reduce the amounts of antibiotics required.

The cranberry fruit is rich in polyphenols, specifically anthocyanins, flavonols and flavan3-ols, which often form oligomeric and polymeric structures, along with lesser amounts of benzoic acid derivatives. These natural compounds in cranberry are often collectively referred to as tannins, which are found in abundance among many plants including berries, leaves, bark and roots. Tannins have many biological activities and flavonoids, including anthocyanins and proanthocyanidins, are believed to be the major antimicrobial components [50]. Here, the relative abundances of constituents from fraction FC111 are described in Table 3. The primary anthocyanins present were peonidin-3-O-galactoside, cyanidin-3-O-galactoside and peonidin-3-O-arabinoside. Malvidin-3-O-galactoside and 3-O-arabinoside were also present, consistent with previously reported literature for both fresh cranberry fruit and juice extracts [33,34]. The dominant flavonol was quercetin-3-O-galactoside (peak 32, 50% abundance), followed by three different quercetin-3-O-pentosides (peaks 34, 35, 36), totaling 25% abundance). While commercial standards were not available for the absolute confirmation of these pentosides, they have been tentatively identified as quercetin-3-O-xyloside, quercetin-3-O-arabinofuranoside, and querecetin-3-O-arabinopyranoside, as previously reported by Borges et al. [33]. Additionally, numerous early eluting peaks (8–16 min) were detected, with absorption wavelength maxima characteristic of benzoic acids (λmax ~280 nm) and hydroxycinnamic acids (λmax ~ 315 nm) (Table 3), also consistent with previously reported literature [33,34]. Characteristic mass spectra for both parent and fragment ions indicates a number of these simple phenolic acids are linked to hexose sugars, but the absolute identity, galactose versus glucose sugar, or exact O-linkage position could not be determined using HPLC-MS methods alone.

From our analysis of the constituents of laboratory-scale fraction FC111, it is unclear, at this point, whether the observed antibacterial activity stems from iridoids, phenolics or flavonoid components, with further isolation and characterization remaining an active area of research. However, the mechanisms of action of our FC111 fraction seem not to be related to its acidic constituents, which negatively impact bacterial proteins and DNA/RNA syntheses [51]. Bactericidal activity of lab-prepared cranberry proanthocyanidins has been reported against MRSA [44]. Proanthocyanidin peaks could not be conclusively identified, based upon the HPLC-MS-conditions used. It is possible that the ethanolic PC extraction may not have been optimal for the solubilization of proanthocyanidins, which are generally extracted with more apolar solvents such as acetone. Given their low (essentially non-abundance) in the extracts studied herein, proanthocyanidins are not expected to play a major role in the observed biological effects, unless extraordinarily active at minute concentrations. Demonstrating novel antibacterial activity from cranberries not associated with proanthocyanidin components is especially exciting, and warrants further investigation.

## Conclusions

Our study explored the mechanism of action of cranberry on *S. aureus* and revealed an antibacterial action associated with the disruption of cell wall biosynthesis similar to other known cell-wall acting antibiotics. Moreover, our results showed the potential therapeutic use of a combination of β-lactams and an easily prepared cranberry extract against *S. aureus* infections such as bovine mastitis.

## Competing interests

The authors declare that they have no competing interests. Also note that Decas Botanical Synergies, the manufacturers of NC90, played no role in the design, implementation, analysis, or financing of this study.

## Authors’ contributions

SB, EB, MG, GB, HR, JH and JM carried out the experiments. MSD, BDO, SB, EB, MG, JH and FM designed and conceived the study. MSD, DBO, SB, JH and FM wrote the paper. All authors read and approved the final manuscript.

## Pre-publication history

The pre-publication history for this paper can be accessed here:

http://www.biomedcentral.com/1472-6882/13/90/prepub
